# Improved spinal fusion efficacy by long-term delivery of bone morphogenetic protein-2 in a rabbit model

**DOI:** 10.3109/17453674.2011.636675

**Published:** 2011-11-25

**Authors:** Jae-Wook Lee, Saehyoung Lee, Sun Hwa Lee, Hee Seok Yang, Gun-II Im, Chang-Sung Kim, Jung-Ho Park, Byung Soo Kim

**Affiliations:** ^1^Orthopaedic Surgery, Korea University Ansan Hospital, Korea University College of Medicine, Ansan; ^2^School of Chemical and Biological Engineering, Seoul National University, Seoul; ^3^Department of Orthopaedics, Dongguk University International Hospital, Goyang; ^4^Department of Periodontology, College of Dentistry, Yonsei University, Seoul, Republic of Korea

## Abstract

**Background and purpose:**

Various new delivery systems for recombinant human bone morphogenetic protein-2 (rhBMP-2) have been introduced to improve its efficacy in osteogenesis. Of these, we have previously developed heparin-conjugated PLGA nanospheres (HCPN) as a long-term delivery system for BMP-2. In vitro studies have shown that the BMP-2 long-term delivery system enhances the level of bone formation. However, the long-term effects of BMP-2 on spinal fusion have not been assessed. Therefore, we now tested the hypothesis that the long-term delivery of BMP-2 using HCPN improves spinal fusion compared to short-term delivery in a rabbit fusion model.

**Methods:**

24 adult New Zealand White rabbits underwent posterolateral fusion (6 animals in 4 groups). The autograft group received an autologous iliac chip bone graft as a positive control. The BMP-2-PN group received rhBMP-2 (20 μg per implant) and PLGA nanospheres (PN) suspended in fibrin gel, and served as a short-term release group. The HCPN group received HCPN suspended in fibrin gel without BMP-2 as a negative control. The BMP-2-HCPN group received rhBMP-2 (20 μg per implant)-bound HCPN suspended in fibrin gel and served as a long-term release group. All animals were killed 12 weeks after surgery. Manual palpation, axial tensile tests, radiography, and histological evaluations were then performed.

**Results:**

The spinal fusion rate and Young's modulus of the fusion mass were better in the BMP-2 long-term delivery group than in the short-term delivery group at an equivalent dose. However, the outcome of the long-term delivery was inferior to that of the autograft group.

**Interpretation:**

The HCPN system showed potential as an effective carrier that might improve the osteogenic efficacy of BMP-2 for spinal fusion.

Recent approaches to regenerate bone have used osteogenic growth factors in combination with carriers or scaffolds (Petite et al. 2000). These factors include bone morphogenic proteins (BMPs), basic fibroblast growth factor (bFGF), and transforming growth factor-β (TGF-β) (Lieberman et al. 2002). However, only BMPs are capable of inducing ectopic bone formation in extra-osseous tissues, and are known to play a key role in osteoinduction. In particular, BMP-2 has been used as osteoinductive growth factor to promote spinal fusion to treat degenerative spondylosis.

BMP-2 and BMP-7 are the two BMPs that have been approved by the US FDA (Food and Drug Administration) for human use to date. The clinical equivalence of BMP-2 in spinal fusions compared to autografts in human trials has been reported. Burkus et al. (2002) reported that treatment of patients with degenerative lumbar disc diseases with recombinant human BMP-2 gave higher interbody fusion rates (95%) than in a control group that received autogenous iliac crest bone graft (89%). Another randomized human clinical study showed that a BMP-2-treated group showed a higher fusion rate (88%) than a control iliac crest bone graft group (73%) in posterolateral fusions (Dimar et al. 2006). In addition, similar results have also been reported regarding the use of BMP-7 in treating posteolateral lumbar arthrodesis (Vaccaro et al. 2008).

A number of different carriers, including synthetic polymers and type-I collagen, have been used to deliver BMP-2 in experimental and clinical studies. However, the results of such attempts have been less than satisfactory, so that no single carrier has been generally accepted as an optimal one (Minamide et al. 2001). Consequently, further effort to develop an optimized system for delivery of BMP-2 for clinical applications is therefore needed.

Recent studies have shown that cell-surface or extracellular matrix-associated polysaccharides, including heparin, can be used for the sustained delivery of BMP-2 to potentiate BMP-2 activity (Takada et al. 2003, Zhao et al. 2006). These findings led to the synthesis of a heparin-conjugated poly(lactic-co-glycolic acid) (PLGA) system for the long-term delivery of recombinant human BMP-2 (rhBMP-2) (Jeon et al. 2007), which was further modified and developed as heparin-conjugated PLGA nanospheres (HCPNs) (Jeon et al. 2008). Based on the direct binding of BMP-2 to heparin, which is known to extend the half-life and bioactivity of BMP-2 (Zhao et al. 2006), HCPNs suspended in fibrin gel have been reported to be effective in bone regeneration by facilitating the long-term delivery of growth factors in vivo, including BMP-2 (Jeon et al. 2006, 2008, La et al. 2010).

To our knowledge, there are no data published on the effects on spinal fusion of the carrier-dependent release pattern of BMP-2. Thus, in the present study, we tested the hypothesis that the long-term delivery of BMP-2 using HCPN would improve spinal fusion compared to the short-term delivery of BMP-2 in a rabbit spinal fusion model. The results of the long-term delivery of BMP-2 using HCPN were compared to those of the short-term delivery achieved through the use of PLGA nanospheres (PNs) without heparin conjugation, in addition to autogenous bone grafts that served as a positive control.

## Material and methods

### Study design

Twenty-four 12-month-old skeletally mature, healthy New Zealand White rabbits (KOATECH, Pyeoungtaek, Korea) weighing 3–3.5 kg were used. Prior to the experiments, the animals were allowed to adjust to their environment for 2 weeks. They were divided into 4 groups (6 per group), based on the implant type. Each animal had a bilateral inter-transverse process arthrodesis between the fifth and sixth lumbar vertebrae using a previously described protocol (Boden et al. 1995). The autograft group received an autogenous iliac chip bone graft and served as a positive control. The BMP-2-PN group received BMP-2 and PNs suspended in fibrin gel without heparin conjugation, and served as a short-term release group. The HCPN group received HCPNs suspended in fibrin gel without BMP-2 and served as a negative control. The BMP-2-HCPN group received BMP-2-bound HCPNs suspended in fibrin gel and served as a long-term release group.

The rabbits were maintained and cared for in accordance with institutional guidelines. The experimental protocol was approved by the Institutional Animal Care and Use Committee (IACUC) of Dongguk University, Goyang.

### Preparation of graft materials

**Figure 1.**
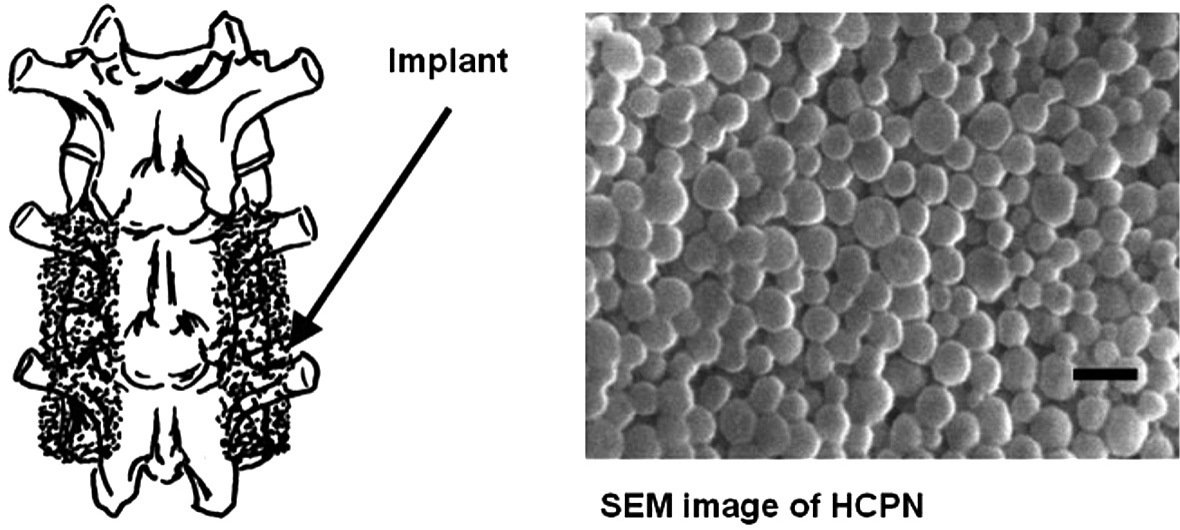
Left panel: Schematic illustration of spinal fusion via implants between the transverse processes of the fifth and lumbar vertebrae. Right panel: Scanning electron microscopic images of HCPN. Scale bar represents 500 nm.

Recombinant human BMP-2 (produced from CHO cells) was purchased from R&D Systems (Minneapolis, MN). Fibrin gel (Greenplast) was purchased from Greencross (Seoul, Korea). PNs (75 mol% of lactide, MW 90,000) and HCPNs (Figure 1, inset image) were manufactured as described previously (Jeon et al. 2006). For the preparation of BMP-2-PNs and BMP-2-HCPNs, BMP-2 (20 μg per implant) was mixed with PNs (50 μg per implant) or HCPNs (50 μg per implant) in fibrin gel (2.5 mL per implant). For the HCPN group, HCPNs (50 μg per implant) were mixed with fibrin gel (2.5 mL per implant) without BMP-2. The morphological examination of HCPNs was performed using a scanning electron microscope.

### Surgery

A posterolateral inter-transverse process arthrodesis was performed in each animal bilaterally between the fifth and sixth lumbar vertebrae. The animals were anesthetized with an intramuscular injection of ketamine (35 mg/kg of body weight) and xylazine (8 mg/kg of body weight) under aseptic conditions. Briefly, in each animal, a dorsal longitudinal incision was made around the spinous process. After fascia exposure, an incision was made using the paramedian approach. The intermuscular plane between the multifidus and longissimus muscles was developed to expose the transverse processes of the fifth and sixth lumbar vertebrae and the inter-transverse membrane bilaterally. Using a 4.0-mm electric burr, the exposed posterior cortex of the transverse process was decorticated and the burred bone chip was irrigated. For the autograft group, an autogenous bone graft from the ileum was obtained through the same skin incision after bilateral fasciotomy on the posterior superior iliac spine. Prepared implants (6 bilateral implants per group), iliac chip bone, BMP-2-PN, HCPN, and BMP-2-HCPN, were grafted between the transverse processes in the paraspinal bed bilaterally (Figure 1). Fascial incisions and the skin were sutured and gentamicin (5 mg/kg) was injected intramuscularly. The animals were housed individually in cages and fed rabbit chow and water ad libitum. The animals were monitored for general health on a daily basis. 12 weeks after surgery, all the animals were killed by barbiturate overdose (100 mg/kg intraperitoneally) without damaging the bilateral fusion masses.

### Assessment of fusion

*Manual palpation.* The fusion mass containing the fifth and the sixth lumbar vertebrae in addition to the spinous processes was protected from external forces during killing. To guarantee a thorough examination of the union, all connective structures between the vertebrae, including the anterior and posterior longitudinal ligaments, the ligaments between the spinous processes, ligamentum flavum, facet joint membrane, and intervertebral disc were completely removed. Gross examination of fusion masses was performed. After the explantation, a manual motion test of intervertebral segments was also performed. Union was defined as being when the continuity was maintained and the flexion and elasticity were similar to the bone tissue. If the fusion masses between the transverse processes displayed flexions resembling those of soft tissue, they were considered to be non-unions. A partial union was defined as being when the results were intermediate between union and non-union.

*Radiographic analysis.* 12 weeks after the surgery, posteroanterior radiographs were taken using a 90-cm tube-to-plate distance. Complete and uninterrupted radiographic bilateral osseous bridging of the transverse processes was considered to be a union. Unilateral osseous bridging or interlaminar bony union was considered to be a partial union, and a lack of ossification or osseous bridging was considered to be a non-union.

*Mechanical testing.* For mechanical testing, all remaining tissues around the bone masses and facet joints were removed, and intervertebral discs were divided with a scalpel so that only the fusion masses and inter-transverse membranes were connected to the vertebrae. Vice-type fixing tools were used to hold both the upper and the lower adjacent vertebral bodies such that the tensile force would be distributed evenly with no rotational stress. The fixing tools were connected to an Instron 8500 (Instron, Norwood, MA) and tensile stress was measured longitudinally. The Young's modulus of each specimen was calculated based on the slope of the tensile stress up to 1 cm/cm strain. Tensile testing was performed on 12 specimens (3 per group).

### Statistics

Quantitative data were expressed as mean (SD). For the statistical analysis on Young's modulus, the one-way ANOVA test with Bonferroni correction was performed using OriginPro 8 SR4 software (version 8.0951, OriginLab Corp., Northampton, MA). For the spinal union data, non-parametric Fisher's exact test was performed using SAS/STAT software. Any p-value less than 0.05 was considered to be statistically significant.

## Results

### Manual palpation

After the surgery, 2 animals were excluded from the HCPN group. 1 died of unknown causes 3 weeks after surgery and the other was killed due to a deep infection. The remaining 22 rabbits tolerated the procedure well and gained weight. After en bloc removal, gross examination of specimens was performed. All specimens were blindly palpated by 2 examiners to assess flexion and extension at arthrodesis levels and at adjacent levels proximally and distally. Gross examination indicated that masses in the BMP-2-HCPN group were larger than those in the BMP-2-PN and HCPN groups. The number of animals showing complete union was highest in the autograft group (5 of 6 animals), followed by the BMP-2-HCPN group (3 of 6 animals) (Table 1). In the other 2 groups, no animals showed complete union. The Fisher's exact test result indicated a correlation between the type of implant and the resultant type of union (p = 0.009).

**Table 1.** Number of animals with different types of unions, as judged by manual palpation

Union type	Autograft	BMP-2-PN	HCPN	BMP-2-HCPN
Complete union	5	0	0	3
Partial union	1	5	2	3
Non-union	0	1	2	0
Total	6	6	4	6

Complete union: fusion mass continuity was maintained and flexion and elasticity were similar to bone tissue Non union: fusion masses displayed flexions resembling those of soft tissue Partial union: intermediate between union and non union Autograft: autogenous iliac chip bone implant BMP-2-PN: BMP-2 (20μg) mixed with PLGA nanospheres (50μg), suspended in fibrin gel (2.5mL) HCPN: heparin conjugated PLGA nanospheres without BMP-2, suspended in fibrin gel BMP-2-HCPN: BMP-2 mixed with heparin conjugated PLGA nanospheres, suspended in fibrin gel Fisher's exact test indicated that there was a correlation between the type of implant and the type of union as a result (p=0.009).

### Radiographic analysis (Figure 2)

In the autograft and BMP-2-HCPN groups, all specimens showed complete bilateral bone bridge formation (6 out of 6 animals for both groups). In the BMP-2-PN group, 2 animals (out of 6) showed complete bone bridge formation and the remaining 4 animals showed incomplete bone bridge formation or unilateral bone bridge formation. In the HCPN group, no complete bone bridge formation was seen (Table 2). The Fisher's exact test result indicated that there was a significant correlation between the type of implant and the resultant type of union (p < 0.001).

**Figure 2.**
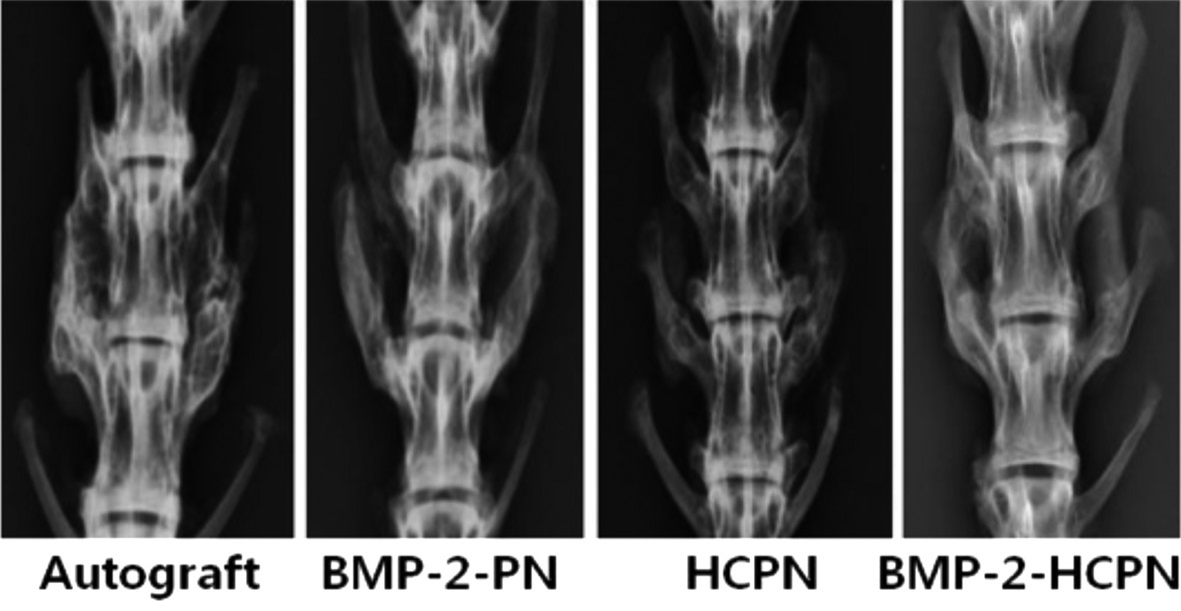
Representative radiographs showing the status of the spinal fusion between the transverse processes of the fifth and sixth lumbar vertebrae in each group.

**Table 2.** Number of animals with different types of unions, as judged by radiographic examination

Union type	Autograft	BMP-2-PN	HCPN	BMP-2-HCPN
Complete union	6	2	0	6
Partial union	0	4	2	0
Non-union	0	0	2	0
Total	6	6	4	6

Fisher's exact test indicated that there was a correlation between the type of implant and the type of union as a result (p=1.789E-04).

### Mechanical testing (Figure 3)

The Young's modulus of the autograft groups was higher than in the other groups (p = 0.01, p = 0.004, and p < 0.001, when compared to autograft, BMP-2, and HCPN, respectively) (Figure 4). Among the other groups, except the autograft group, the BMP-2-HCPN had a statistically significantly higher Young's modulus than the BMP-2-PN group or the HCPN group.

**Figure 3.**
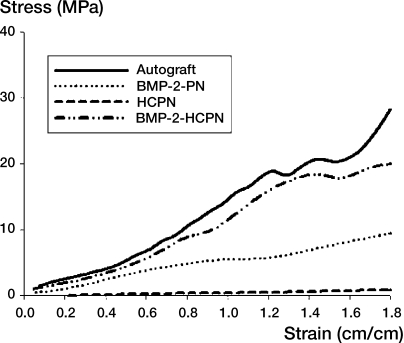
Representative stress-strain curves obtained by axial tensile testing. Longitudinal tensile stress of the 2 adjacent vertebral bodies (fifth and sixth) was measured using vice-type holders connected to a measuring device.

**Figure 4.**
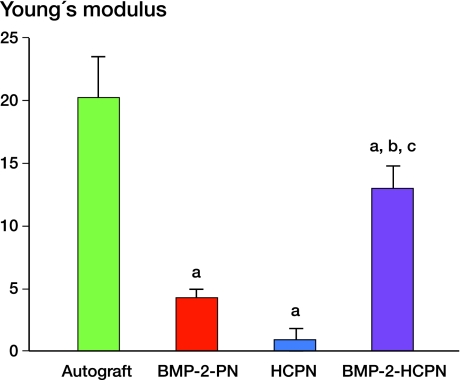
Young's modulus determined by the result of axial tensile testing. Values represent mean (SD). **^a^** p < 0.05, compared to the autograft group; **^b^** p < 0.05, compared to the HCPN group; and **^c^** p < 0.05, compared to the BMP-2-PN group.

## Discussion

We found that at an equivalent dose, the HCPN-mediated long-term delivery of BMP-2 resulted in a higher number of complete spinal fusions compared to the short-term delivery in a rabbit model, demonstrating the importance of delivery systems or carriers of BMP 2 in inducing bone regeneration. Due to the rapid local clearance and short biological half-life of BMP-2 (Ruhe et al. 2006), high doses of BMP-2 are required to induce spinal fusion, i.e. its routine clinical use is not cost-effective (Hsu and Wang 2008). Modification of carriers or delivery systems can enhance the efficacy of local BMP-2 delivery (Akamaru et al. 2003, Liao et al. 2003); thus, efforts to optimize BMP-2 delivery systems have been made and evaluated. We have previously reported that heparin-conjugated PLGA systems, including scaffolds or nanospheres, can prolong the release of BMP-2 at the target site (Jeon et al. 2007, 2008, La et al. 2010). In these delivery systems, the fibrin gel allows the HCPNs to be suspended so that they can be injected in a minimally invasive manner, and the high surface-to-volume ratio of the nanospheres allows large amounts of BMP-2 to be loaded (Jeon et al. 2008).

In the present study, the long-term delivery of BMP-2 using HCPN resulted in a higher spinal fusion rate and improved mechanical properties compared to the short-term delivery or the negative control (HCPN) group. Since the only variable between the BMP-2-HCPN group and the BMP-2-PN group was the heparin conjugation, the higher spinal fusion rate of the BMP-2-HCPN group appears to have been due to the presence of heparin in the delivery system. In this heparin-conjugated PLGA system, BMP-2 directly binds to heparin, inhibiting BMP-2 degradation and prolonging the half-life of BMP-2 (Zhao et al. 2006). Long-term delivery of BMP-2 using HCPN in combination with fibrin gel has been reported to enhance orthotopic bone formation compared to short-term delivery (La et al. 2010). These studies indicate that the heparin-conjugated PLGA delivery systems release bioactive BMP-2 over a prolonged period of time and more efficiently induce bone formation.

Although the ability of BMP-2-HCPN to induce bone formation was better than either BMP-2-PN or HCPN, it was still not as effective as the autograft. Furthermore, the fusion strength was higher in the autograft group than in the BMP-2-HCPN group. A possible reason for the inferiority of the BMP-2-HCPN group to the autograft group may have been the low dose of BMP-2 we used. Ectopic bone formation is one of the most frequently reported adverse effects of using BMP-2 or BMP-7, and using a minimal dose of BMPs has been suggested as one of the possible solutions (Benglis et al. 2008). Consequently, to minimize the possibility of ectopic bone formation, we used a relatively small amount of BMP-2 (20 μg per implant) compared to that used in other previous studies using a similar delivery strategy. For example, a previous study showed that 3 mg of BMP-2 in combination with nano-hydroxyapatite/collagen/polylactic acid (nHAC/PLA) achieved lumbar spinal fusion comparable to that from autogenous iliac crest in rabbits (Liao et al. 2003). However, our data indicate that the dose of BMP-2 we used might be too low to produce comparable bone formation to that from autograft.

In summary, the number of complete spinal fusions was higher in the BMP-2 long-term delivery group using HCPN than in the short-term delivery group at an equivalent dose in vivo. However, probably due to the low dose of BMP-2 used in this study, the results of the long-term delivery group were inferior to those in the autograft group. Our findings suggest that HCPN could be a useful carrier for BMP-2 and might improve the osteogenic efficacy of BMP-2 for spinal fusion in vivo. Additional studies are required to optimize the BMP-2 dose.
